# Advances in 3D Bioprinting and Microfluidics for Organ-on-a-Chip Platforms

**DOI:** 10.3390/polym17223078

**Published:** 2025-11-20

**Authors:** Natan Roberto de Barros, Samarah Vargas Harb, Cintia Delai da Silva Horinouchi, Larissa Bueno Tofani, Daniela Mayra dos Santos, Giovanna Blazutti Elias, Julia Carnelos Machado Velho, Ana Carolina de Aguiar, Monielle Sant’Ana, Ana Carolina Migliorini Figueira

**Affiliations:** National Laboratory of Bioscience (LNBio), National Center of Research in Energy and Materials (CNPEM), Campinas 13083-100, Brazil; natan.barros@lnbio.cnpem.br (N.R.d.B.); samarah.harb@lnbio.cnpem.br (S.V.H.); cintia.horinouchi@lnbio.cnpem.br (C.D.d.S.H.); larissa.tofani@lnbio.cnpem.br (L.B.T.); daniela.santos@lnbio.cnpem.br (D.M.d.S.); giovanna.elias@lnbio.cnpem.br (G.B.E.); julia.velho@lnbio.cnpem.br (J.C.M.V.); ana.aguiar@lnbio.cnpem.br (A.C.d.A.); monielle.leal@lnbio.cnpem.br (M.S.)

**Keywords:** 3D bioprinting, bioinks, microfluidics, organ-on-a-chip, tissue engineering

## Abstract

The convergence of 3D bioprinting and microfluidics has revolutionized the development of organ-on-a-chip platforms, offering unprecedented opportunities in biomedical research and tissue engineering. This comprehensive review delves into the latest advancements in these technologies, highlighting their significance and transformative potential. The introduction provides an overview of 3D bioprinting, microfluidics, and organ-on-a-chip systems, emphasizing their critical roles in replicating physiological conditions and enhancing the precision of biomedical studies. The review aims to move beyond fundamental concepts, focusing on recent innovations and applications that have propelled these technologies to the forefront of research. In the realm of 3D bioprinting, the review explores the evolution of bioprinting techniques, including extrusion-based, inkjet, and laser-assisted methods and polymer-based biomaterials as matrices for in vitro tissue modeling. Technological breakthroughs such as high-resolution bioprinting, multi-material printing, and advanced bioink development are discussed, showcasing their impact on creating complex tissue structures. Innovations in bioinks, including printable polymer-based hydrogels and decellularized matrix bioinks, are highlighted for their ability to replicate tissue microenvironments more accurately. The review also covers microfluidic innovations, detailing advances in design and fabrication, including 3D printing and sensor integration. Key innovations in fluid dynamics and tissue integration are examined, demonstrating how these advancements enhance tissue modeling and mimic physiological perfusion. Developing multi-organ-on-a-chip systems and connecting multiple tissue types for systemic studies are also explored. Hence, integrating 3D bioprinting and microfluidics is a focal point, with discussions on how their convergence enhances organ-on-a-chip platforms. The review concludes by examining current challenges, such as scalability and regulatory hurdles, and future directions, including emerging technologies like 4D bioprinting and AI-driven tissue design.

## 1. Introduction

The rapid advancements in biomedical research and tissue engineering have been significantly propelled by innovative technologies, including 3D bioprinting, microfluidics, and organ-on-a-chip (OoC) systems [1,2,3]. These technologies have revolutionized the way researchers approach the study of human physiology, disease modeling, and drug testing, offering unprecedented precision and control over the replication of complex biological systems [4].

3D bioprinting is a cutting-edge technology that involves the layer-by-layer deposition of bioinks to create three-dimensional structures that mimic the architecture and functionality of natural tissues [5]. This technique allows for the precise placement of cells, biomaterials, and soluble factors, enabling the fabrication of complex tissue constructs with high spatial resolution [6]. The significance of 3D bioprinting in biomedical research lies in its ability to produce tissue models that closely resemble their in vivo counterparts, providing a more accurate platform for studying cellular interactions, disease progression, and therapeutic responses [7,8,9].

Microfluidics, on the other hand, involves the manipulation of fluids at the microscale level, typically within channels that are tens to hundreds of micrometers in diameter [10,11]. This technology enables the precise control of fluid flow, allowing for the creation of dynamic microenvironments that can mimic the physiological conditions of tissues and organs [12]. Microfluidic devices, often referred to as “lab-on-a-chip” or “organ-on-a-chip” systems, have been widely used in various applications, including diagnostics, drug delivery, and tissue engineering [13]. The integration of microfluidics with 3D bioprinting has further enhanced the capabilities of these technologies, enabling the development of more sophisticated and functional tissue models [13,14].

OoC systems represent a convergence of biofabrication methods, including 3D bioprinting and microfluidics, offering a powerful platform for replicating the structure and function of human organs in vitro [6,15]. These systems consist of microfluidic channels embedded with tissues, bioprinted or not, allowing for the precise control of the cellular microenvironment and the simulation of physiological processes such as nutrient transport, waste removal, and mechanical stimulation [16,17]. Currently, these systems have been set up on diverse platforms, which have emerged as valuable tools for studying organ-level functions, disease mechanisms, and drug responses, providing a more accurate and ethical alternative to traditional animal models [18,19,20].

**Figure 1:**
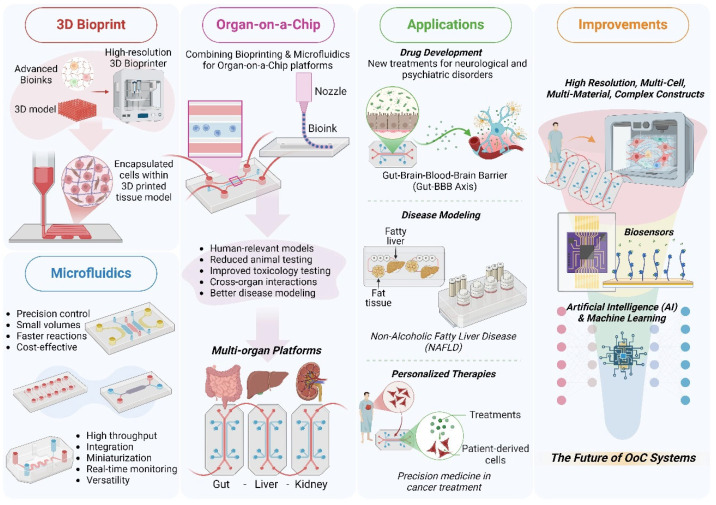
Integrated Overview of 3D Bioprinting and Microfluidics for OoC Platforms and Applications. This schematic illustrates the pivotal role of 3D bioprinting, highlighting components such as advanced bioinks designed for biocompatibility and precision, 3D models enabling high-resolution tissue architecture, and cutting-edge bioprinters capable of encapsulating living cells. Together, these innovations create the foundation for physiologically relevant tissue models. Complementing this, microfluidic systems offer precision control, reduced sample volumes, faster reaction times, and high-throughput capabilities, with miniaturized designs adaptable for diverse biomedical applications. Integrating 3D bioprinting and microfluidics enables advanced OoC platforms that reduce reliance on animal testing, enhance disease modeling, and simulate cross-organ interactions, exemplified by multi-organ systems connecting the gut, liver, and kidney. Applications include drug development, such as modeling the Gut–Brain–Blood–Brain Barrier (Gut-BBB Axis) for neurological treatments, disease modeling with Non-Alcoholic Fatty Liver Disease (NAFLD) platforms, and personalized therapies using patient-derived tumor cells for tailored cancer treatments. Future advancements aim to integrate high-resolution bioprinting with biosensors, AI, and machine learning (ML), promising a new era of automation, optimization, and predictive capabilities for biomedical research and therapeutic innovation. Created in BioRender. Figueira, A. (2025). https://www.biorender.com/8ti0pdy.

Here, we provide a comprehensive overview of recent innovations and applications in 3D bioprinting and microfluidics for OoC platforms, highlighting the transformative potential of integrating these technologies into biomedical research and tissue engineering (Figure 1). It delves into advancements in bioprinting techniques, including high-resolution, multi-material printing and bioink development, such as printable hydrogels and decellularized matrix bioinks which enhance tissue microenvironment replication. Progress in microfluidic design, including 3D-printed devices, sensor integration, and multi-OoC systems, is also examined, showcasing improved modeling of physiological perfusion and systemic studies. The review explores the synergy of bioprinting and microfluidics, detailing co-culture systems and dynamic tissue interactions for enhanced tissue maturation and differentiation. Applications in drug discovery, disease modeling, and personalized medicine are discussed, along with examples of OoC models advancing research on cancer, fibrosis, and neurodegenerative diseases. Finally, it addresses current challenges, such as scalability and regulatory hurdles, highlighting future directions involving 4D bioprinting, artificial intelligence (AI)-driven design, and interdisciplinary collaboration to propel innovation and broaden clinical and industrial adoption.

Hence, this review provides a comprehensive understanding of the recent advancements in 3D bioprinting and microfluidics for OoC platforms. By highlighting the most significant technological breakthroughs and their applications, this review underscores the transformative potential of these technologies in biomedical research and tissue engineering.

## 2. Technological Innovations in 3D Bioprinting

### 2.1. Evolution of Bioprinting Techniques

**Figure 2:**
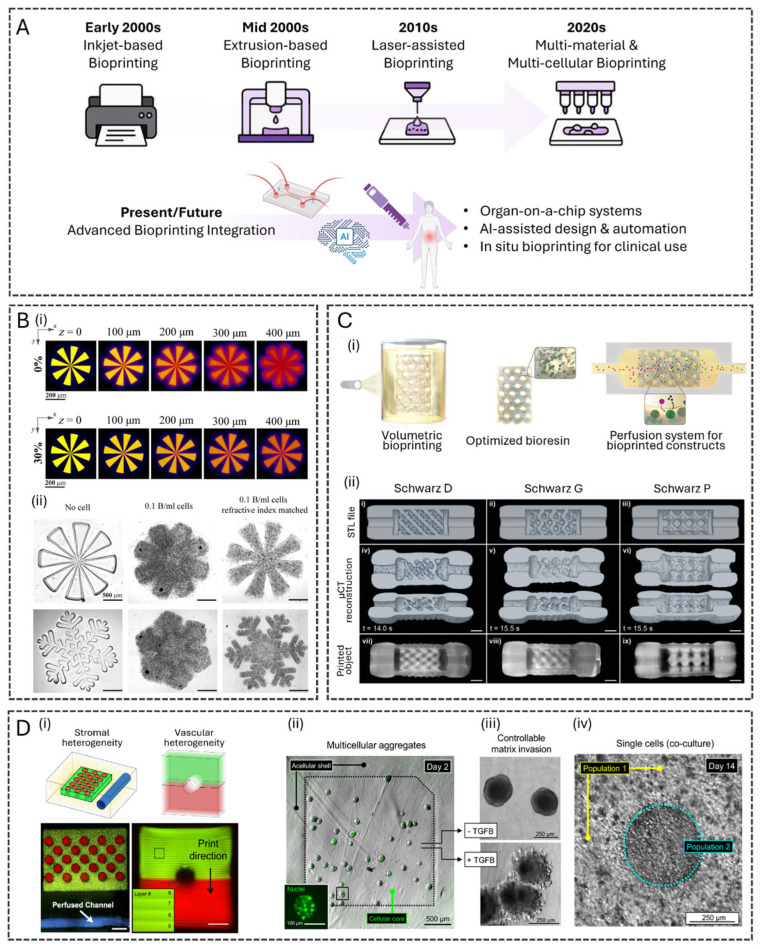
(**A**) Evolution of 3D Bioprinting. Schematic illustrating the evolution of 3D bioprinting technologies over time. It begins with simple inkjet adaptations in the early 2000s, progresses through extrusion and laser-based techniques, and leads to current multi-material printing. The future points toward integrated systems with AI, OoC, and in situ clinical applications. Created in BioRender. Figueira, A. (2025) https://BioRender.com/pfg19v5. (**B**) High cell density and high-resolution DLP–based 3D bioprinting with iodixanol-added bioinks. (**i**) Projected pattern at different z depths of the cell-encapsulated bioink with 0% and 30% iodixanol. (**ii**) Printing resolution comparison among three different bioink compositions: bioink without cells, bioink with 0.1 billion cells/mL, and refractive index–matched bioink with 0.1 billion cells/mL. Scale bars, 500 μm. Reproduced from [28], with permission from The American Association for the Advancement of Science, 2023. (**C**) High-resolution VBP. (**i**) Representation of the volumetric printing process of a complex, organoid-laden printed biofactory cultured under dynamic perfusion to enhance hepatic function. (**ii**) (i–ix) Complex, perfusable architectures printed with a lattice design that enables coupling to microfluidic tubing (scale bars = 2 mm). Reproduced from [32], with permission from Wiley, 2022. (**D**) Multi-material SLA printing of heterogeneous structures. (**i**) Hydrogel features with encapsulated fluorescent beads. All scale bars = 1000 μm. (**ii**) Direct bioprinting of pre-formed multicellular aggregates. Inset: a green fluorescent protein (GFP)-tagged nuclear transcription factor enables single-cell visualization. (**iii**) Aggregates cultured only in growth media maintained their original spherical morphologies, while aggregates exposed to hTGF-β1 adopted invasive morphologies. (**iv**) An idealized model of intratumoral heterogeneity. By day 14, the separate cell populations grew to form a dense and seamless regional interface, likely suitable for longitudinal interrogation of emergent tumor mosaicism. Reproduced from [33], with permission from Springer Nature, 2021.

3D bioprinting has evolved through a series of technological advancements, encompassing four primary methods: inkjet-based, extrusion-based, laser-assisted, and stereolithography (SLA) bioprinting (Figure 2A) [21]. Starting in the early 2000s with adapted inkjet printers, the field progressed to extrusion-based systems in the mid-2000s for improved material handling and cell viability [22]. The 2010s saw the introduction of laser-assisted techniques for high precision and nozzle-free printing, followed by light-based methods such as SLA for rapid, high-resolution fabrication. Today, these technologies form a robust and versatile toolbox for engineering functional tissue models, enabling researchers to select the most suitable approach for their specific biological and structural requirements.

Extrusion-based bioprinting, the most widely used technique in the field, relies on the continuous deposition of cell-laden bioinks through a nozzle, allowing for controlled layer-by-layer construction of large, complex structures [23]. With a resolution of 100 to 500 µm, extrusion-based bioprinting can print high-viscosity bioinks and build stable, large-scale constructs, making it accessible and versatile for many laboratories [24]. However, cell viability is moderate due to shear stress, which can vary depending on factors such as the viscosity of the bioink, the printing speed, the nozzle diameter, and the applied pressure. Key innovations in extrusion-based printers include temperature-controlled printheads for thermosensitive bioinks, advanced pneumatic and screw-driven dispensing mechanisms, and software-driven extrusion profiles to achieve complex geometries and gradient structures. These features help ensure consistent cell viability and structural integrity, even for large or thick tissue constructs. Advanced extrusion-based bioprinters have also incorporated multiple print heads, enabling multi-material deposition.

Inkjet bioprinting uses droplets of bioink ejected by thermal or piezoelectric forces, enabling high-resolution patterns with relatively gentle handling of cells. This technique typically achieves resolutions around 100 to 500 µm and ensures excellent cell viability, making it suitable for detailed, cell-rich constructs [25]. However, it is limited to low-viscosity bioinks and is less effective for creating large structures. Innovations like precise droplet volume control, enhanced speed, and the integration of real-time monitoring have significantly improved reproducibility and reliability in tissue model fabrication using inkjet technology.

Laser-assisted bioprinting employs focused laser energy to transfer small volumes of bioink onto a substrate, achieving fine precision and cell placement [26]. Among the highest resolution methods, often below 10 µm and allowing single-cell placement, laser-assisted bioprinting also provides cell viability exceeding 95%. However, the technique is costlier, more complex, and slower overall. Advances in laser optics and beam control have enhanced resolution and patterning speed, allowing more intricate tissue constructs to be fabricated faster. Moreover, integrating automated calibration systems and improved user interfaces has streamlined the workflow, reduced complexity, and made the process more accessible to researchers.

SLA has recently emerged as a promising bioprinting method. This technique uses a focused laser or digital light projection (DLP) to crosslink photopolymerizable bioinks layer-by-layer, creating highly detailed structures with resolutions down to 10 µm. SLA allows for high precision and smooth surface finishes, making it particularly effective for printing intricate vascular networks and complex scaffolds. Cell viability typically ranges from 70 to 90%, with outcomes depending on the careful optimization of light intensities and exposure times to minimize cellular damage [27]. However, the main drawbacks of SLA include a narrower range of compatible bioinks, limited to photopolymerizable materials, and potential challenges in achieving uniform cell distribution within the construct. In addition, bioprinting resolution deteriorates as bioink cell density increases, due to light scattering. When light passes through a bioink containing cells, the difference in refractive index between the cells and the surrounding material causes significant scattering, blurring the projected light, and reducing printing resolution. A strategy to mitigate this effect using iodixanol has been reported [28], where the biocompatible compound works by tuning the refractive index of the bioink to match that of the encapsulated cells’ cytoplasm, thereby reducing light scattering. By incorporating iodixanol, scattering effects decrease by approximately 10-fold, achieving a 50 µm resolution in a bioink with a cell density of 0.1 billion cells per milliliter (Figure 2B). Advances in SLA comprise the introduction of multi-wavelength light sources and advanced curing strategies. These tools have recently allowed the creation of gradient structures and more complex, multi-material constructs.

A specialized light-based bioprinting technology, known as volumetric bioprinting (VBP), has gained increasing attention. VBP enables the rapid formation of entire 3D structures within seconds by projecting a holographic or tomography-like light field into a bioink-filled vat, inducing volumetric crosslinking at specific regions [29,30]. This non-layered approach eliminates the need for mechanical movement between layers, resulting in higher resolution, smoother surfaces, and faster fabrication. Additional advantages of VBP include the absence of shear stress and reduced light exposure compared to SLA, leading to improved cell viability [31]. The potential of VBP for high-throughput fabrication of liver organoids for in vitro drug testing has been highlighted [32]. Centimeter-scale 3D structures were successfully printed using optical tomography in under 20 s (Figure 2C). The gelatin methacryloyl (GelMA) resin was supplemented with iodixanol to counteract cell-mediated light scattering and enhance printing resolution. The printed hepatic organoids, derived from intrahepatic bile duct stem cells, demonstrated high viability, hepatocytic differentiation, and metabolic activity, including albumin synthesis and ammonia detoxification when cultured under dynamic perfusion conditions.

Multi-material bioprinting allows for the simultaneous deposition of different cell types, matrix components, and functional biomaterials [33]. This capability supports fabricating heterogeneous tissues, such as skin models with distinct epidermal and dermal layers or organoids with multiple cellular zones [34]. A notable example of multi-material bioprinting was reported for corneal tissue engineering [35]. Using extrusion-based bioprinting, alternating bioinks were deposited to mimic the native lamellar structure of the corneal stroma, where collagen fibrils are arranged in perpendicular layers. The authors utilized two hyaluronic acid (HA)-based bioinks with different stiffnesses: a soft, cell-laden bioink and a stiffer, acellular bioink. The soft bioink supported cell proliferation and tissue formation, while the stiff bioink provided structural integrity and guided cellular organization.

In an innovative strategy, an SLA bioprinter was integrated with a microfluidic mixer chip to produce either continual or discrete gradients of bioinks [36]. Using multiple inlets and a microfluidic channel, the bioinks are mixed and dispensed in a vat where the construct is printed. This system eliminates the need for washing steps between ink exchanges, reducing bioink waste and improving fabrication efficiency. The researchers confirmed the device’s efficiency in achieving precise control over cell density, growth factor concentration, hydrogel stiffness, and porosity gradients in both horizontal and vertical directions.

Another multi-material SLA bioprinting innovation was developed to fabricate heterogeneous structures while preventing unwanted bioink mixing [33]. The semi-automated printer integrates an automated material selection process with a manual saline rinsing step. Using this bioprinter, the authors successfully constructed monolithic, multi-material hydrogels with discrete cellular and acellular domains, enabling controlled spatial heterogeneity (Figure 2D). The system was validated using fluorescent tracers and morphometric image analysis, demonstrating seamless bioink integration. The study also highlights potential biological applications, including the bioprinting of lung adenocarcinoma cells in core/shell architectures to investigate tumor–stromal interactions and intratumoral heterogeneity modeling.

By using multiple bioinks in a single construct, researchers can mimic the complexity of native tissues, facilitating more physiologically relevant studies of development, disease, and regeneration. Further advancing these capabilities, integrating coaxial and triaxial nozzles enables the controlled deposition of multiple bioinks in intricate configurations.

These innovations have significantly enhanced bioprinting reliability, scalability, and reproducibility. As a result, they have facilitated the creation of more precise tissue models, opening the door to bioprinted tissues that more closely replicate physiological function and structure.

### 2.2. Advanced Bioinks for 3D Bioprinting

Advances in bioinks have enhanced bioprinted tissue fidelity by optimizing both composition and rheological and mechanical properties. Improved formulations provide a supportive scaffold that promotes cell viability, proliferation, and differentiation, leading to more functional and biomimetic tissues [17,37]. Among the materials explored for bioink formulation, decellularized extracellular matrix (dECM) has gained attention for its ability to retain native extracellular matrix (ECM) components, including growth factors and signaling molecules, thereby improving tissue-specific functions [5].

A bioink composed of alginate and dECM extracted from the lung has been reported, which can be used to bioengineer robust structures at anatomically relevant scales for human tissue [38]. The study evaluated the angiogenic potential of the bioink both in vitro and in vivo. The results showed that the hydrogel containing dECM promoted the growth of new blood vessels. In in vivo tests on rats, the implant was well integrated into the surrounding tissue after implantation of the biomaterial, with no evident signs of inflammation. Vascularization was more pronounced in dECM hydrogels, which supported an intact vascular network throughout the entire thickness of the graft. As proof of concept, the authors demonstrated that bioinks containing lung dECM could be used to 3D bioprint a human subsegmental bronchus, composed of primary pulmonary smooth muscle cells and primary human airway epithelial progenitor cells.

Natural biomaterials such as collagen, gelatin, HA, fibrinogen, silk, gellan gum, dextran, agarose, chitosan, and alginate [39] have been widely used due to their biocompatibility, tunable viscosity, and ability to mimic the native cellular microenvironment [37]. A bioink incorporating gelatin, alginate, HA, and fibrinogen was developed to support tissue regeneration [39]. In this formulation, gelatin enhances printability and provides structural support; alginate ensures mechanical stability; HA contributes lubrication and hydration; and fibrinogen promotes cell adhesion. This combination supports tenocyte viability over three weeks, with notable proliferation during the first 7 days and stabilization between days 14 and 21. In vitro assays revealed enhanced capillary-like loop and tube formation in human umbilical vein endothelial cells (HUVECs) cultures with VEGF165 (vascular endothelial growth factor 165) release, and increased proliferation and viability with PDGF-BB (platelet-derived growth factor-BB).

Integrating natural and synthetic biomaterials continues to refine bioink formulations, balancing mechanical properties with biological factors. Modifying natural materials is widely used in bioink formulation. For instance, an ionic-photo-crosslinkable hydrogel was engineered by methacrylation of gelatin from porcine skin (GelMA) and further optimization with 2D nanosilicates (Laponite) to improve the hydrogel’s rheological and mechanical properties [17]. By leveraging the ionic charge properties of Laponite, the engineered bioink enhances the retention of biological factors secreted by encapsulated cells, promoting paracrine signaling and improving the physiological relevance of in vitro tumor models. The study systematically evaluates the printability and mechanical properties of GelMA–Laponite bioinks, demonstrating that variations in GelMA and Laponite concentrations influence cell behavior. Notably, higher Laponite content leads to enhanced cell aggregation and upregulation of growth factor and tissue remodeling-related genes in tumor cells, while also inducing a mesenchymal phenotype in fibroblasts.

Given the advancements in bioink formulation, there has been a continuous evolution in the search for compositions that balance mechanical, printability, and biological properties, enabling the creation of more suitable microenvironments for bioprinting complex tissues. Combining natural and synthetic biomaterials, performing chemical modification of polymers, and incorporating bioactive and ceramic components have expanded the possibilities in tissue engineering. Hence, the choice of the ideal bioink still depends on the specific requirements of each application and biocompatibility, reinforcing the importance of personalized strategies for the bioprinting of functional structures.

### 2.3. Bioprinting for Complex Tissue Structures

Bioprinting techniques have advanced to the point where highly sophisticated tissue constructs, including vascularized networks, perfusable channels, and multi-layered assemblies, can be reliably produced [40]. These developments enable more accurate, functional models of human physiology, bridging the gap between engineered tissues and their natural counterparts.

**Figure 3:**
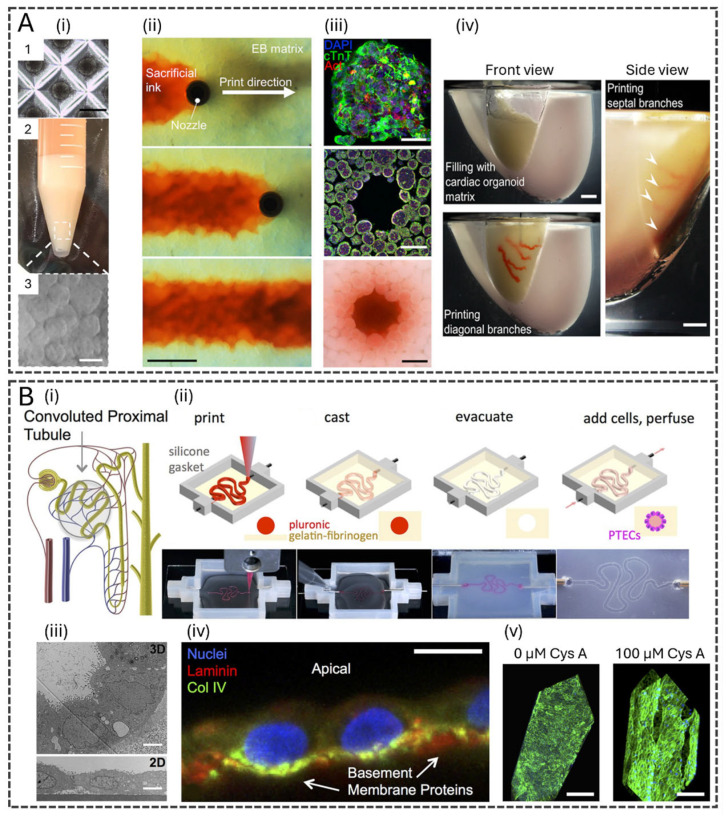
Bioprinting for Complex Tissue Structures. (**A**) Tissues with high cellular density and embedded vascular channels. (**i**) (1) Microwell culture of embryoid bodies (EB)-based OBBs. Scale bar, 300 μm. (**ii**,**iii**) OBB tissue matrix. Scale bar, 200 μm. (**ii**) Time-lapse of sacrificial ink (red) writing via embedded 3D printing within an EB matrix. (**iii**) Example of an OBB-based matrix composed of cardiac spheroids. Row 1: Individual OBB with characteristic markers. Rows 2 and 3: Cross sections of immunostained slices and bright-field images, respectively, of the OBB. Scale bars, 50 μm (top row) and 500 μm (middle and bottom rows). (**iv**) A 1:2 scale polydimethylsiloxane mold is formed, and the left anterior descending artery, together with diagonal and septal (arrowheads) branches are embedded into a septal-anterior wall wedge of the cardiac tissue matrix. Scale bar, 5 mm. Reproduced from [43], with permission from The American Association for the Advancement of Science, 2019. (**B**) 3D convoluted renal proximal tubule on a chip. (**i**) Schematic of a nephron highlighting the convoluted proximal tubule, (**ii**) Corresponding schematics and images of different steps in the fabrication of 3D convoluted, perfusable proximal tubules: a fugitive ink is first printed on a gelatin–fibrinogen ECM, additional ECM is cast around the printed feature, the fugitive ink is evacuated to create an open tubule, and PTECs are seeded within the tubule and perfused for long periods. (**iii**) Transmission electron microscopy (TEM) image of the PTECs within the tubule at 5 weeks and PTECs grown on a 2D dish coated with ECM with no perfusion, scale bar = 5 μm. (**iv**) PTECs at 6 weeks showing the basement membrane proteins the cells secreted, scale bar = 10 μm. (**v**) 3D renderings of 3D convoluted renal proximal tubule on chips after drug treatment, scale bars = 200 μm. Reproduced from [46], with permission from Springer Nature, 2016.

Vascularization represents a particularly critical challenge and opportunity. Recent studies have demonstrated the successful bioprinting of microvascular networks that support perfusion, nutrient delivery, waste removal, and dynamic biochemical communication between cells [34,41,42]. Different techniques have been employed to create tubular structures lined with endothelial cells, achieving structural integrity and functional endothelialization. For instance, an extrusion-based technology using a sacrificial gelatin ink was described [43]. The sacrificial gelatin was printed within a densely cellular matrix composed of collagen I and Matrigel loaded with induced pluripotent stem cell (iPSC)-derived organ building blocks (OBBs). The process of forming tubular structures is achieved by printing the gelatin ink at low temperature as the core component of a core–shell coaxial bioprinting, followed by warming the printed constructs to 37 °C, which melts and evacuates the sacrificial gelatin ink, leaving behind interconnected channels (Figure 3A). The study successfully demonstrated the functionality of the vascularized tissue by fabricating perfusable cardiac tissues that mature and beat synchronously.

Also, for cardiac applications, researchers have employed 3D bioprinting to achieve thick and perfusable cardiac patches [44]. dECM was extracted from human omental tissue, and the extracted cells were reprogrammed into pluripotent stem cells, following differentiation into cardiomyocytes and endothelial cells. Cardiomyocytes were embedded in a thermoresponsive dECM hydrogel, while endothelial cells were incorporated into a sacrificial gelatin bioink. Using an extrusion-based 3D bioprinter with dual print heads, cardiac patches were fabricated, simultaneously depositing cardiac and vascular bioinks. Liquefying the gelatin bioink at 37 °C created perfusable vascular channels lined with endothelial cells. Functional assessments confirmed synchronized cardiomyocyte contraction and efficient electrical signal propagation. When transplanted into rat omentum, the patches integrated successfully with the host tissue.

The formation of vascular channels has also been explored in bone tissue engineering. A noteworthy example utilized VBP to fabricate bone-like tissues within seconds [45]. The bioink consisted of GelMA, human mesenchymal stem cells (hMSCs), and HUVECs. The HUVECs self-organized into an endothelium-lined channel and promoted osteogenic differentiation, as evidenced by the increased expression of early osteocytic markers (PDPN—podoplanin, DMP1—dentin matrix acidic phosphoprotein 1).

The fabrication of perfusable channels by 3D bioprinting has also been used to replicate tissues where controlled fluid dynamics are essential, such as in kidney tubules, bile ducts, and gastrointestinal tracts, enabling the study of transport mechanisms, drug absorption, and waste filtration in biomimetic models. A notable study bioprinted human renal proximal tubules on perfusable chips, providing an advanced kidney tissue model for drug screening and disease modeling [46]. Using extrusion-based bioprinting with a sacrificial Pluronic ink core, the authors created tubules with an open lumen, embedded within a gelatin–fibrin matrix (Figure 3B). The printed tubules were seeded with proximal tubule epithelial cells (PTECs) and maintained under physiological shear stress, leading to enhanced cell polarity, brush border formation, and functional transporter expression. The bioprinted tubules exhibited long-term viability (>60 days), improved epithelial morphology, and barrier function, outperforming traditional 2D culture models. Additionally, the system was validated through nephrotoxicity assays using Cyclosporine A, demonstrating its potential for predicting drug-induced kidney toxicity.

Extrusion-based bioprinting approach has been employed to fabricate a tubular 3D intestine-on-a-chip [47]. Using a triaxial nozzle, this method enabled the precise deposition of three distinct bioinks to create a perfusable, multi-layered tubular structure: (1) an alginate–collagen bioink containing smooth muscle cells (HSISMC), (2) a collagen bioink containing vascular endothelial cells (EA.hy926), and (3) a sacrificial gelatin bioink encapsulating intestinal epithelial cells (Caco-2). Upon bioprinting, the gelatin bioink liquefied at physiological temperature, facilitating the formation of a hollow lumen. By integrating dynamic culture conditions, the model successfully replicated key physiological features, such as villi and crypt formation, mucus secretion, and barrier function, outperforming traditional 2D transwell models.

Another key area of innovation lies in the development of multi-layered tissue structures. Bioprinting methods now enable the assembly of stratified tissues, such as skin models with distinct epidermal, dermal, and hypodermal layers. A full-thickness human skin equivalent was printed with hypodermis (HSEH) using a collagen-based bioink in a cooled extrusion-based printhead [48]. Integrating a hypodermal layer yielded a complex tissue structure resembling native human skin and the expression of numerous genes essential for skin function, including those related to hydration, development, and differentiation. Another multi-layered bioprinting strategy for skin tissue engineering was reported, with a mobile in situ bioprinting system that can deposit dermal fibroblasts and epidermal keratinocytes within a collagen/fibrin hydrogel onto an injured area [49]. The system integrates 3D imaging to precisely map wound topography and an inkjet-based bioprinter for controlled cell deposition. Murine and porcine wound models demonstrated significantly accelerated wound closure, reduced contraction, enhanced re-epithelialization, and improved tissue organization compared to conventional treatments, highlighting the system’s potential for personalized wound healing applications.

Multi-layered 3D bioprinting has also enabled the creation of biomimetic liver tissue by integrating human induced pluripotent stem cell (hiPSC)-derived hepatic progenitor cells with endothelial and stem cells [50]. Using SLA-based technology and bioinks composed of GelMA and glycidyl methacrylate hyaluronic acid (GMHA), researchers were able to produce patterns that mimic the hepatic lobule structure [50]. The authors reported enhanced liver functions closely associated with the 3D assembly of hepatocytes, improved morphological organization, elevated liver-specific gene expression, and increased metabolic product secretion compared to 2D culture.

In addition to skin and liver models, other studies have explored multi-layered 3D bioprinting to fabricate complex tissues, including cardiovascular [50], intestinal [51], and corneal [35] structures. These multi-layered constructs mimic the heterogeneity of native tissues, recapitulating the intricate cellular architecture and functional specialization found in vivo. By incorporating multiple cell types in spatially organized layers, these constructs can replicate key physiological processes, such as parenchymal and stromal interactions, zonal metabolic gradients, and organ-specific signaling pathways [17].

Overall, the application of bioprinting for complex tissue structures redefines the scope of OoC systems. Three-dimensional tissue models are moving closer to replicating the structure and function of native tissues, offering unprecedented opportunities for biomedical research and therapeutic innovation. These complex tissues are foundational for OoC systems, facilitating long-term cell culture and dynamic flow conditions.

## 3. Microfluidic Innovations for Organ-on-a-Chip

### 3.1. Advances in Microfluidic Design, Fabrication, and Tissue Integration

Microfluidic systems are broadly defined as platforms that manipulate and control small volumes of fluids, typically in the range of nanoliters to microliters, within channels whose characteristic dimensions usually fall between tens and hundreds of micrometers [13,52]. Such scales ensure that laminar flow, diffusion-dominated transport, and precise spatiotemporal gradients prevail, enabling accurate mimicry of the microenvironmental conditions found in living tissues [53]. However, the term “microfluidic” is used pragmatically in the OoC field to encompass devices with millimeter-scale channels fabricated by accessible methods such as laser cutting or soft-lithography with poly(methyl methacrylate) (PMMA) or polydimethylsiloxane (PDMS), since they still exhibit fluid dynamics characteristic of microscale systems and support essential biological functions such as nutrient perfusion, shear-stress control, and barrier maintenance [53]. These versatile fabrication strategies facilitate integration with sensors, membranes, and tissue scaffolds, thereby bridging the gap between engineering precision and biological relevance. Overall, microfluidic innovations have been central to the evolution of OoC platforms, providing physiologically relevant flow control and enabling high-throughput modeling of organ-level physiology and pathology.

Recent developments in microfabrication techniques have highlighted the importance of comparing three commonly employed lithography-based methods in microfluidics: SLA, photolithography, and soft lithography. All three rely on light-based patterning, but differ in mechanisms, resolution, and typical wavelength ranges. SLA, widely used in 3D printing of microfluidic structures, employs UV or near-UV light (~385–405 nm) to photopolymerize liquid resins layer by layer [54,55]. Photolithography, the traditional method in semiconductor and microfluidic master fabrication, uses mask-based exposure of photoresists to deep-UV light (e.g., 248–193 nm), achieving sub-micron feature resolution [56]. Soft lithography, often used for PDMS-based devices, involves creating an elastomeric master (via photolithography or laser-based methods) followed by replica molding. While highly flexible and cost-effective, soft lithography generally produces larger features, ranging from a few micrometers to millimeters [57].

These lithography techniques provide a foundation for advanced 3D bioprinting approaches, which enable the precise layer-by-layer construction of complex microfluidic devices using computer-aided design (CAD) software [1]. Selecting appropriate materials is crucial, favoring transparency, biocompatibility, elasticity, and cost-effectiveness [58]. Among 3D printing methods, SLA and DLP have gained prominence due to their micrometer-scale resolution in the XY plane and rapid fabrication speed [59]. SLA relies on a UV laser to crosslink biomedical-grade resins, whereas DLP uses a UV projector and an array of micromirrors, often employed in PDMS reverse molding techniques.

Integrating biosensors, chemosensors, and metallic microcapillary arrays within microfluidic platforms has expanded their functionality, enabling real-time measurements of key biological parameters such as oxygen levels, lactate, glucose, and extracellular field potentials [60]. Additionally, transepithelial electrical resistance (TEER) is frequently utilized to assess barrier integrity, which is essential for maintaining tissue homeostasis [61].

OoC technologies have been increasingly adopted for high-throughput drug screening, allowing the simultaneous testing of multiple drug concentrations on hundreds of 3D cell cultures within a single chip [62]. For instance, a large-scale phenotypic screening approach using a 3D angiogenesis-on-a-chip assay has been demonstrated, which enables the evaluation of over 1500 kinase inhibitors and identifies compounds with desirable efficacy and toxicity profiles [63]. Similarly, a programmable 96-well OoC system with integrated fluid flow and real-time sensors has been developed, allowing multiplexed culture of human tissues and high-content analysis under dynamic conditions [64]. In another study, a reusable microfluidic chip capable of combinatorial drug screening on tumor spheroids was introduced, showcasing its potential for assessing drug efficacy and synergistic interactions in a high-throughput manner [65]. These examples illustrate the growing relevance of OoC technologies as scalable and physiologically relevant platforms for drug discovery.

Despite the success of commercial devices such as TissUse’s Humimic Starter and Emulate’s Zoe, challenges remain regarding scalability and reproducibility [66]. To address these issues, high-throughput screening platforms are incorporating laboratory automation, AI, and standardized regulatory frameworks to ensure the consistent fabrication of OoC models [67].

Efficient microfluidic design is essential for maintaining cell viability and tissue function by ensuring continuous nutrient and oxygen delivery [68]. Perfusion plays a critical role in sustaining long-term tissue culture, with flow regulation systems necessary for various OoC models, including kidney, skin, liver, lung, and blood–brain barrier (BBB) chips [69]. To optimize perfusion, microfluidic platforms often integrate pumping systems equipped with flow sensors for active flow rate monitoring and regulation [70]. Computational modeling further enhances precision and cost-effectiveness by optimizing flow dynamics within the system [71].

Beyond sustaining cellular viability, microfluidic devices facilitate inter-organ communication, enabling advanced pharmacokinetic and drug metabolism studies [72]. The integration of multiple tissue types within a single platform allows researchers to better replicate in vivo physiological interactions [73]. In this context, 3D bioprinting continues to revolutionize microfluidic technology by enabling the fabrication of both complex microfluidic architectures and biomimetic 3D cell cultures, paving the way for next-generation biomedical research and therapeutic applications [74].

Single-channel chips were used to evaluate the influence of cystic fibrosis transmembrane conductance regulator (CFTR) on insulin secretion. With dimensions 0.14 mm (thickness), 26.87 mm^2^ (area), and 3.76 mm^3^ (volume), it was designed to mimic pancreatic duct-like structures, fabricated by standard photolithography and soft lithography. In this device, pancreatic ductal epithelial cells were cultured in the top chamber while pancreatic cells were in the bottom chamber. After CFRT inhibition, a reduced insulin secretion was observed (53.7%; Δ191.4 μLU/mL) from islet cells. In double-channel chips from islet cells without pancreatic ductal epithelial cells (PDECs), the CFTR inhibition did not show a change in insulin secretion, while in both single and double pancreatic-on-a-chip, higher insulin secretion was induced after the high-glucose exposition (450 mg/dL) [75].

3D Organoid-derived PTECs (OPTECs)-on a chip from human pluripotent stem cells (hPSCs) differenced into nephron progenitor cells and PTECs were fabricated using a channel template composed of fishing line (100–380 µm in diameter) in polycarbonate, It was secured onto a metal base plate with two 50 m × 75 mm glass slides using 9 M4 12 mm screws, being added gelatin–fibrinogen matrix into each chip to provide an enzymatic crosslink. A medium reservoir was projected using a 10 cc syringe barrel connected to a 0.2 µm syringe and, two-stop Ismatec peristaltic tubes were attached to the syringe nozzle used to connect the outlet pins to silicone tubing that returns media to 10 cc reservoirs. OPTECs-on a chip were more sensitive to predicting drug nephrotoxicity (cisplatin and aristolochic acid) compared to traditional models from immortalized cells, once it provides enhancement in perfusion on proximal tubular segments within kidney organoids [76].

A gut–liver-on-a-chip has been designed to study NAFLD. The NAFLD-chip was made of PDMS membrane (200 × 200 µm^2^ area and 20 µm thickness) using a multilayer soft lithography replica molding technique, with two cell cultures (220 µm height and 2.1 mm width) linked by microfluidic channels (45 µm height and 2.1 mm width), and a pump with positive hydraulic pressure (150 kPa). The pressure was controlled and operated by a pneumatic system using a controller board. Caco-2 and hepatocellular carcinoma cells (HepG2) were introduced into individual cell culture chambers with a medium circulation flow of 15 nL min^−1^ for 7 days. To induce NAFLD, a mix of Palmitic Acid (PA) and Oleic Acid (OA) was performed in two treatment times (1 and 7 days), representing initiation and progression of the disease. After 7 days, lipid accumulation was observed in mono and co-culture Caco-2:HepG2, associated with reduced apoptosis and an increase in albumin expression, a functional hepatocyte marker, suggesting liver functionality improvement after microfluidic display. In the case of Caco-2, PA, and OA treatments after 1 day, DNA damage was initiated due to cellular disruption by copper ions. In contrast, in 7 days, an increase in apoptotic cells was observed in co-culture Caco2:HepG2. When evaluating the effects of NAFLD induction on co-culture Caco:HepG2, mRNA-seq results showed elevated expression of genes related to mitosis in Caco-2 cells. In contrast, HepG2 cells demonstrated upregulation of genes involved in the cell cycle. The developed platform demonstrated accurate results in establishing a new drug for NAFLD and other disorders associated with the gut–liver axis [77].

A BBB-on-a-chip was fabricated using PDMS using soft lithography with three channels: upper, center, and lower (400 µm, 300 µm, and 200 µm, respectively). A polycarbonate membrane (8 µm pore) treated with 5% 3-aminopropyl-triethoxysilane (APTES) solution was bonded between the upper and lower PDMS layers using a plasma cleaner. Human brain vascular pericytes (HBVP) were added into the center channel (10^7^ cells/mL) onto a fibronectin-coated polycarbonate membrane. Human astrocytes (10^6^ cells) with Matrigel solution were added to the same channel as HBVP cells. After 6 h at 37 °C, human brain microvascular endothelial cells (HBMEC) (7 × 10^7^ cells) were seeded into the fibronectin-coated upper channel. After 24 h, the upper channel was connected to syringe pumps (16 µL/min) under shear stress dyne cm^−2^, corresponding to brain conditions. The BBB-on-a-chip device demonstrated tight barrier integrity with a permeability coefficient according to the in vivo tissue, and recapitulation of BBB organization, integrity, and physiology. As in the in vivo-like morphology, the BBB-on-a-chip device demonstrated reduced astrogliosis markers, as LCN2 (lopocalin 2) expression, indicating the important value of the model in neuroinflammatory diseases. Polarized distribution of AQP4 (aquaporin 4) demonstrated the potential for modeling reactive gliosis in Central Nervous System (CNS) diseases, once it maintains water and ion homeostasis in the brain. High-density lipoprotein (HDL)-mimetic nanoparticles showed the ability to cross the BBB via SR-B1 (scavenger receptor class B type 10), mediated transcytosis, reinforcing the relevance of BBB-on-a-chip development in the drug delivery area [78].

A human heart-on-a-chip comprising cardiomyocytes, vascular endothelial cells, and fibroblasts was fabricated to mimic in vivo cardiac behavior. In this study, a commercial Emulate Chip S1 fabricated with PDMS was used as a model, being designed by a rectangular upper channel (1 mm width, 1 mm height, and 17 mm length), a lower channel (1 mm width, 0.2 mm height, and 17 mm length), and a thin film (50 µm) separating them. The chip surface was coated with Matrigel, seeding HUVECs into the bottom channel. After 8 days in perfusion culture, a mix of 1 × 10^5^ iPSCs and 1.7 × 10^5^ human gingival fibroblasts (HGFs) was added into the upper channel. iPSCs–fibroblasts–endothelial chip exhibited sensitivity to drug response to noradrenaline and nifedipine, and better recapitulation with human heart function than isolated cells. Followed by increased cardiomyocyte marker cardiac troponin t (cTnT) and ventricular cardiomyocyte marker IRX4, and enhanced contractibility of iPSC provided by fibroblasts, heart-on-a-chip demonstrated potential use to investigate human cardiac drug toxicity [79].

### 3.2. Microfluidics for Multi-Organ Systems

Single-organ-on-chip models enable a detailed study of each organ’s unique characteristics, responses, and functions. In contrast, multi-organ platforms integrate dynamic interactions between organs and tissues, offering a more comprehensive understanding of physiological processes and systemic reactions [80].

Multi-organ-on-chips (multi-OoC) are miniaturized microfluidics-based platforms comprising multiple chambers, each representing an organ or tissue. Hence, mimicking interactions and reactions between organs inside a single system that produces a dynamic environment closely resembling the architecture of the human body [81,82]. Therefore, multi-OoC platforms can adequately facilitate systemic approaches for predicting the response of several organs under specific conditions, which makes the system an attractive platform for studying organ interactions, drug testing/screening, and disease modeling in vitro [53,82,83].

As an illustration of that, a simple all-PDMS microfluidic device was constructed to investigate the impact of hepatocyte metabolites on intestinal permeability [84]. Using the microphysiological system, researchers could observe that Caco-2 permeability decreased when in co-culture with human hepatic progenitor cells (HepaRG).

Similarly, a gut–liver axis multi-OoC device has been developed to evaluate cellular fluidic shear stress and inter-tissue interaction by integrating two cell-culture chambers, enabling different and physiologically relevant flow perfusion to each organ. The results evidenced that under the physiologically relevant flow, the Caco-2 and HepG2 cells maintained a cell survival rate of 95% and 92%, respectively, and that the expression of functional proteins of each organ was enhanced [85].

To explore the relationship between the gut environment and the brain’s neurocognitive functions, a modular Gut–Brain Axis (GBA) chip containing gut and BBBs was developed [86]. Increased permeability of both barriers was observed throughout TEER analysis after administering lipopolysaccharide (LPS), an inflammatory microbial byproduct. On the other hand, their integrity was improved when sodium butyrate, known to enhance the gut’s epithelium barrier function and exert a neuroprotective effect, was applied to the gut barrier module. Moreover, cells of both tissues seemed less sensitive to LPS in transwell conditions than in on-chip conditions, suggesting that the flow may affect their response. Furthermore, the transport of fluorescently labeled exosomes across the gut barrier towards the BBB was observed. Additionally, the presence of flow increased the exosome uptake by the mono-cultured brain endothelial cells (hBMECs). In light of these results, the fluidic condition appears to enhance exosome transport across the gut epithelium and uptake by the brain endothelial cells.

Besides studying organ interactions, the multi-OoC devices are interesting alternatives in the process of drug development since they enable the analysis of drug metabolism, excretion, absorption, and toxicity in vitro [53,82]. For instance, Ferrari et al. developed a multi-OoC platform to study off-target cardiotoxicity of liver-metabolized drugs. To enable that, the device contained, in one chamber, a metabolically competent liver model, which was evidenced to produce albumin at 7 days of culture. The hepatic model was connected through microchannels to a functional 3D heart model, in a second chamber, in which the cardiac cells were spontaneously beating after 3 days and expressing typical cardiomyocytes and gap-junction markers after 7 days of culture (troponin and Cx43, respectively) [87]. Moreover, LivHeart predicted Terfenadine’s off-target cardiotoxicity more physiologically than available single-organ microfluidic platforms.

In another study, a hepatic cellular model was connected to 3D ovarian cancer tissues to investigate drug efficacy and hepatotoxic effects of antineoplastic drugs in vitro [88]. The 3D ovarian cancer models showed higher drug resistance than the 2D models in static conditions. Also, in comparison to 2D and 3D static models, the experimental approach combining 3D culture, fluid-dynamic conditions, and multi-organ connection was revealed as the most predictive of toxicity and efficacy results compared to clinical therapy.

As an alternative platform to evaluate toxicological endpoints for cosmetic ingredients and other chemicals, an OoC microfluidic device with skin, intestine, and liver equivalents was developed. Known toxicants (acetaminophen and formaldehyde) were applied in the skin compartment as topical treatments to validate the model. The modulation of gene expression in each organ equivalent was measured, demonstrating the model’s effectiveness, thus presenting it as a reliable and ethical method for safety assessment in the cosmetic industry [89].

In another study, the same multi-organ platform was later used to evaluate the effects of Bisphenol A (BPA) and Bisphenol S (BPS) via topical and oral administration. Evaluating gene markers associated with carcinogenicity, systemic toxicity, and endocrine disruption, BPA showed expected absorption, leading to liver damage and genetic changes. In contrast, BPS, though considered safer, also affected gene expression, particularly in topical absorption. These findings highlight the need for further investigation into BPA and BPS toxicity and the importance of OoC technology in assessing health risks [90].

Moreover, it has been evidenced that multi-OoC can be applied to investigate the pharmacokinetics of drug–drug interactions (DDIs) [91]. Aiming to assess OoC systems as DDI studies platforms, a multi-OoC device containing a liver compartment as the metabolic model and a lung cancer compartment as the drug target model has been constructed. Besides that, a pharmacokinetic-pharmacodynamic (PK-PD) mathematical model was developed to describe the system. The study evaluated the anticancer drug CPT-11 and assessed DDI through the evaluation of the inhibitory effects of simvastatin (SV) and ritonavir (RTV) on its metabolism using the multi-OoC device and comparing it with the PK-PD model [91]. Both mathematical and experimental models presented similar results, suggesting that combining the PK–PD model and the Multi-OoC is a useful way to predict DDI.

Additionally, multi-OoC systems have been used as platforms to model complex diseases involving multiple organs. A model connecting hepatocytes and adipose tissue compartments was constructed to investigate the metabolic factors contributing to NAFLD development and progression. Their results indicated that the adipocyte module significantly affected NAFLD’s progression in hepatocytes, evidenced by insulin-resistant biomarkers, differential adipokine signaling, and increased tumor necrosis factor-alpha (TNF-α)-induced steatosis [92].

Complementarily, a three-organ device was designed to recapitulate glucose metabolism and homeostasis [81]. First, a model of two chambers containing skeletal muscle (C2C12 myoblasts) and pancreatic tissues was constructed. Based on the measurements of glucose uptake by the muscle cells and insulin secretion by the pancreas cells, a mathematical model of glucose metabolism was developed and used to predict the concentration profiles of glucose and insulin in the muscle, pancreas, and blood chambers in the Multi-OoC. However, the results obtained (glucose and insulin profiles) did not match data from the literature. Therefore, the size of the organ chambers, cell density, and the flow rate can affect the concentration profile of molecules in a multi-organ system. The device design was modified to obtain a more physiological scaling ratio between the muscle and pancreas chambers. After the modifications, the new parameters were applied to the mathematical model of the muscle-pancreas device, resulting in closer, but still different profiles from human physiological values. To overcome that, a liver compartment was added, recognizing its essential role in glucose metabolism, as responsible for producing glucose and the clearance of insulin. The new predictions from the mathematical model showed that glucose and insulin concentration profiles matched those reported in the literature, suggesting that adding the liver to the mathematical model made the glucose and insulin profiles more physiologically realistic. However, the concentration of insulin in the blood in the multiorgan device differed from that in humans, evidence that new optimizations are still needed [81].

Despite recent advancements in multi-OoCs, several challenges remain to be addressed. One critical aspect in developing physiologically relevant multi-systems is selecting an appropriate culture medium, as different organs and tissues have distinct metabolic and nutritional requirements. In this regard, identifying a universal medium capable of sustaining complex multicellular networks is essential [80]. Moreover, studies such as those conducted by Yang et al. are crucial since, physiologically, different organs are submitted to differential flow rates and share stress perfusions [85].

Furthermore, physiological barriers like skin, gastrointestinal tract, lungs, and BBB necessitate distinct engineering strategies and perfusion techniques compared to parenchymal tissues such as fat, kidney, heart, liver, and pancreas, which are more effectively modeled using 3D culture approaches [88]. As an interesting alternative, 3D bioprinting has gained prominence in OoC engineering, significantly developing more accurate and physiologically relevant models.

### 3.3. Quantitative Performance of 3D Bioprinting and Microfluidic Organ-on-a-Chip Systems

Recent experimental studies have increasingly reported quantitative performance indicators for 3D bioprinting and microfluidic OoC technologies, allowing a more objective evaluation of their progress. Table 1 summarizes representative data from the latest literature, highlighting parameters such as printing resolution, cell viability, perfusion flow rate, and barrier integrity metrics. Extrusion-based and VBP techniques typically achieve feature resolutions between 50 and 500 µm, with post-printing cell viabilities ranging from 80 to 95% [32,43,45]. Light-based SLA approaches have reached even higher precision (down to 10 µm) with moderate cell viability (70–90%), depending on photo-exposure control [27]. In microfluidic OoCs, physiological shear stresses of 0.1–10 dyn/cm^2^ are routinely maintained to sustain endothelial function [78,85], while barrier models such as the blood–brain barrier or intestinal epithelium report trans-endothelial electrical resistance (TEER) values within the in vivo-like range of 150–300 Ω·cm^2^ [78,93]. These quantitative metrics collectively demonstrate the significant maturation of 3D bioprinting and microfluidic systems toward reproducing native tissue physiology.

### 3.4. Technological Synergies: Bioprinting Meets Microfluidics

3D bioprinting and microfluidics are complementary technologies that have significantly advanced the development of complex tissue models and OoC systems. While 3D bioprinting enables precise spatial control over cell placement, biomolecules, and biomaterials, microfluidics facilitates the manipulation of fluids at the micron scale, allowing for controlled flow dynamics and biochemical gradients. Combining these two technologies can contribute to different matters in constructing complex tissues. The two main approaches to constructing OoC systems combined with 3D bioprinting are the two-step and the single-stage manufacturing methods.

The two-step approach involves printing micro-organs separately and assembling them onto prefabricated microfluidic platforms [80]. As an example of that approach, a 3D microfluidic-bioprinted model of a vascularized glioblastoma multiforme (GBM)-on-a-chip was designed, aiming to replicate the pathophysiological conditions of the tumor and surrounding vascular microenvironment in vitro. In the first step, the MPS was constructed in PDMS with a photolithography-made mold. In the second one, the tumor tissue comprising highly concentrated GBM cells and surrounding brain endothelial cells was bioprinted directly into the tissue compartment of the PDMS device [42].

The two-step approach has also been used to construct a physiologically relevant human alveolar lung-on-a-chip model. The lung model was first printed inside culture inserts, using inkjet-printing technology, and then the inserts were implanted into a biochip with a flow of culture medium [94].

However, the two-step approach still relies on manual intervention, which can lead to difficulties in automation, inconsistencies in reproduction, and a higher risk of contamination. In contrast, the single-stage approach fabricates the entire chip in a single process, including cells, mechanical components, and microfluidic channels, thus improving automation and scalability [80]. Employing the single-stage approach, an OoC platform was constructed in which diverse cell types and biomaterials were successfully tested and positioned at the desired position for various applications. A liver model was selected to evaluate the developed method, and the liver function was significantly enhanced on the liver-on-a-chip prepared by 3D bioprinting [95]. In another example, a multi-material DLP bioprinting method was used to construct one-step prototyping of hydrogel-based microfluidic chips [96]. From a general point of view, both methodologies have advantages and disadvantages. While the two-step process increases the likelihood of errors due to manual intervention and the greater number of steps involved, it allows the use of conventionally fabricated microfluidic chambers and channels with high resolution. On the other hand, the one-step fabrication of the device via 3D printing undermines the resolution of the final model. There are additive manufacturing methods, such as soft lithography, that allow a higher resolution fabrication; however, these methods are not applicable for multi-material printing involved in the one-step fabrication approach [2]. Thus, two-step and single-stage approaches have shown promising results in developing organs-on-a-chip using 3D bioprinting technology. However, further investigation is required to optimize the printing process, enhance automation, and ensure reproducibility and scalability [80].

### 3.5. Co-Culture Systems and Dynamic Tissue Interactions

Co-culture systems are methodologies that combine two or more cell types in a single culture environment. These systems investigate interactions between different cell types found in human tissues and organs, where the cells can be seeded directly together or separately. Scaffolds can be employed to create a conducive microenvironment that supports cell survival, development, and intercellular communication [97,98,99].

Integrating bioprinting technologies and OoC systems has significantly advanced co-culture models. These technologies enhance architectural precision, reproducibility, and dynamic fluid flow, creating an optimal environment for studies requiring long-term cell viability and functionality [100]. For instance, a human alveolar lung-on-a-chip model was developed using inkjet bioprinting of four alveolar cell types [94]. The model featured a PDMS insert-mountable chip, enabling lung tissue growth at the air-liquid interface. The alveolar barrier model was constructed in culture inserts by sequentially depositing lung endothelial cells, fibroblast-containing collagen, and two types of human alveolar epithelial cells using a drop-on-demand inkjet bioprinter. The resulting tissue, with a thickness of about eight μm, closely mimicked the human alveolar barrier, outperforming prior models with a thickness of >200 μm, limited due to biofabrication methods with reduced precision and automation. The tissues within the microfluidic model exhibited robust tight junctions, evidenced by increased TEER and tight junction protein (ZO-1) expression, with enhanced gene expression of pulmonary function-related genes compared to 2D models, highlighting its physiological relevance.

Similarly, 3D bioprinting has been pivotal in studying the tumor microenvironment. Tumors are inherently heterogeneous, varying in cell types, density, and spatial localization. A 3D-bioprinted tumor-on-chip model of breast cancer was constructed to investigate cell migration in response to the chemoattractant epithelial growth factor (EGF) [101]. The model utilized triple-negative malignant breast cancer cells (MDA-MB-231) and non-tumorigenic mammary epithelial cells (MCF10A) bioprinted with alginate (4%) and gelatin (4%) bioinks. Exposure to an EGF gradient for 8 h revealed critical insights into tumor architecture and cell migration dynamics. Three different cell proportions were used to construct the tumor microenvironment within the microfluidic device. The highest migration was observed when cancerous and non-cancerous cells were in similar proportions. In contrast, migration decreased in environments with a higher number of cancer cells, and was negligible when MCF10A cells predominated. This co-culture bioprinted model thus provided valuable insights into cellular behavior during metastasis. Despite these advancements, co-culture systems face notable limitations. Each cell type often requires a distinct medium composition, making the selection of a universal culture medium challenging [102]. Additionally, the presence of extracellular factors secreted by one cell type can influence neighboring cells, altering medium supplement concentrations [103]. Addressing these issues requires the implementation of biosensors to monitor and maintain essential medium characteristics, such as metabolite levels, within physiological limits. This integration could pave the way for more complex, reproducible, and physiologically relevant in vitro tissue models [102].

### 3.6. Fluidically Enabled Tissue Maturation and Differentiation

Dynamic fluidic flow acts as a biomechanical stimulus that modulates cellular responses in the luminal and interstitial space while facilitating the transport of essential nutrients and signaling molecules [104,105]. Fluid flow stimulates cells through shear stress (tangential to the cell surface) and press stress (normal to the cell surface) [104]. The cellular response to these biomechanical stimuli depends on the duration, magnitude, and frequency, which can induce changes in morphology, orientation, polarization, migration, adhesion, and differentiation [105,106]. In this context, OoC systems stand out as platforms where the fluid flow provides these biomechanical stimulations, besides permitting the replication of vascular perfusion, interstitial flow, circulating immune cells, and critical pharmacokinetic processes such as absorption, distribution, metabolism, and excretion (ADME) [107]. These capabilities have made OoC systems increasingly popular for drug-screening applications [106].

With an extrusion-based coaxial bioprinting approach, Song et al. developed a microfluidic, cylindrical, small intestine-on-chip model composed of three layers: smooth muscle cells, endothelial cells, and epithelial cells [47]. Compared to a 2D transwell dynamic culture containing only epithelial cells, the bioprinted model integrated with microfluidics exhibited significant growth in villi height, particularly under dynamic flow conditions, confirming its ability to support well-structured villi formation. Additionally, this dynamic bioprinted model demonstrated significantly higher expression of MUC-2, a glycoprotein essential for mucus production in the small intestine, and ZO-1, a tight junction protein indicative of barrier integrity, highlighting the improved differentiation and functionality of cells in a dynamic microfluidic environment.

Computational fluid dynamics (CFD) is a powerful tool for analyzing shear stress distribution on cells, which plays a crucial role in enhancing differentiation and functional tissue maturation. In microfluidic systems, CFD facilitates the assessment and optimization of device performance concerning fluid dynamics [108]. This approach enables the simulation of key parameters, including geometry, design, meshing, initial-boundary condition settings, execution of the solver, and results from analysis [109]. Widely used CFD software packages include ANSYS FLUENT and COMSOL Multiphysics, which enable researchers to model complex flow patterns, pressure gradients, and shear stress distributions within microfluidic channels [71,110]. These tools allow the simulation of laminar and transient flow regimes, multiphase interactions, and solute transport, providing insights that guide the optimization of channel geometry, flow rate, and material selection for improved cellular responses and tissue maturation [93,111]. For instance, the effects of shear stress on Caco-2 cells across different stages of polarization were investigated using CFD simulation [93]. The study demonstrated that in a gut-on-a-chip, cell polarization and microvilli formation were strongly influenced by shear stress, with dynamic conditions enhancing the expression of differentiation markers and promoting the development of crypt-villi structures.

Therefore, combining 3D bioprinting, microfluidics, and CFD contributes to developing more physiologically relevant models, enhancing the reproducibility and scalability of bioengineered tissues.

## 4. Applications in Biomedical Research and Therapeutics

### 4.1. New Approach Methodologies (NAMs) and Organ-on-a-Chip for Ethical and Effective Drug Discovery

One of the most prominent and groundbreaking applications of OoC technology lies in developing new approach methodologies (NAMs), which have become a cornerstone in advancing the discovery and evaluation of new drugs and healthcare products. NAMs encompass any technology, methodology, strategy, or combination thereof that provides improved insights into the safety or toxicity of chemical compounds for human health while contributing to eliminating the need for animal testing [112]. Efforts focus on optimizing safety assessment by refining existing methods rather than developing entirely new ones. Strategic repurposing of established methodologies can qualify as NAMs, exemplifying a paradigm shift toward reducing reliance on animal models. Consequently, NAMs can be categorized, albeit in a simplified and non-comprehensive manner, into three principal domains: chemical, silico, and in vitro methods [113]. Within the in vitro domain, the development of 3D-bioprinted/OoC platforms represents a cutting-edge and promising innovation [114]. Three-dimensional Bioprinted/OoC systems, as a NAM, provide human-based, non-clinical models with significant advantages over animal models. Beyond addressing ethical concerns, they offer superior predictive accuracy for human physiological responses. Animal models often lack translational relevance, whereas OoC platforms improve safety assessments and expedite the development of health products, particularly in toxicity screening and preclinical research [115].

Scientific evidence supports the potential of OoC technology as a revolutionary advancement in the pharmaceutical industry [116]. In recent years, beyond the development of new platforms, the establishment of robust assays and standardized protocols has played a pivotal role in building confidence in the technology. This gradual progress is steadily paving the way for integrating OoC systems into the routine workflows of pharmaceutical industries, enabling more informed decision-making and supporting various stages of drug development [117].

The first major milestone in this unstoppable shift in drug development is the Food and Drug Administration (FDA)’s approval of a clinical trial for an antibody developed by the immunology healthcare company Sanofi, based on data generated from a complex human-on-a-chip platform. This groundbreaking decision marked the first time a regulatory agency accepted efficacy data from this technology as sufficient evidence to proceed to human trials, bypassing the need for prior animal testing. Representing a crucial development for rare diseases like chronic inflammatory demyelinating polyneuropathy and multifocal motor neuropathy, which cannot be replicated in animal models [118]. More recently, the FDA announced the acceptance of the Liver-Chip into a validation process to evaluate and qualify the technology for assessing drug-induced liver injury during drug development (DDT-IST-000016). Although not yet formally recognized by regulatory agencies, many companies are actively developing and already integrating OoC platforms into their workflows for toxicity screening, drug testing, and ADME studies [119,120,121]. Driven by the European Medicines Agency’s (EMA) roadmap to phase out animal testing by 2030, several pharmaceutical companies have accelerated the adoption of these technologies as ethical and predictive alternatives [122]. AstraZeneca, for instance, has incorporated advanced microphysiological systems, including OoC and organoids, into its workflows, alongside AI and in silico modeling, to improve predictions of drug safety and efficacy. Similarly, Sanofi utilized efficacy data generated from a human-on-a-chip model developed by Hesperos to support a phase 2 clinical trial of the monoclonal antibody riliprubart (SAR445088), one of the first instances where the FDA accepted a clinical trial application based primarily on OoC-derived data. These initiatives underscore a broader shift in the pharmaceutical industry toward innovative, animal-free research platforms in response to evolving regulatory and ethical standards [123,124].

### 4.2. Disease Modeling and Precision Medicine

Advances in the regulatory acceptance of OoC technologies highlight their transformative potential in biomedical research and drug development (DDT-IST-000016). Microfluidic models address the limitations of animal models and conventional 2D or 3D cultures by replicating complex physiological environments [125]. These platforms provide critical insights into disease mechanisms while reducing high costs and failure rates in drug development [126]. Leveraging diverse cell sources, including stem cells, patient-derived cells, or immortalized cell lines, OoCs have been used to study diseases driven by pathogens [127], lifestyle factors [128], or genetic predispositions [129].

**Figure 4:**
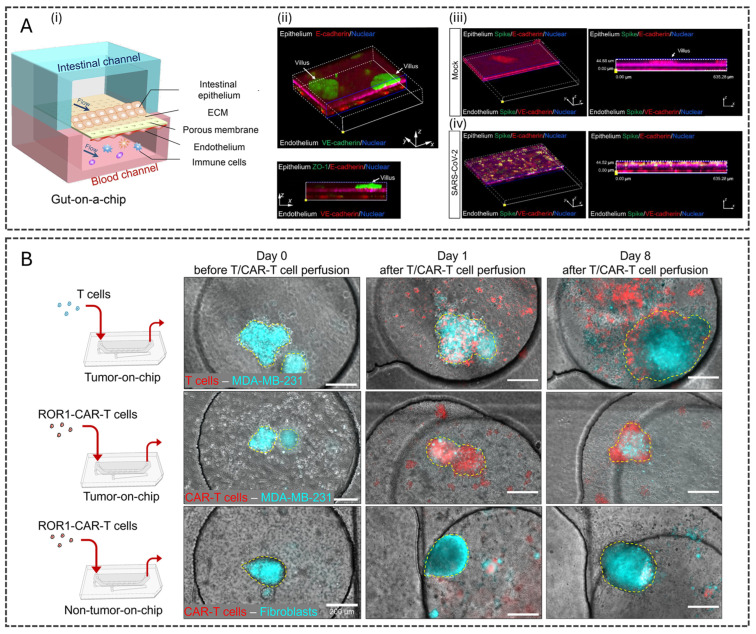
Disease Modeling and Precision Medicine. (**A**) SARS-CoV-2 induced intestinal responses with a biomimetic human gut-on-chip. (**i**) Schematic of the multilayered intestine on the chip device infected with SARS-CoV-2. (**ii**) 3D reconstructed confocal image and side view of the intestinal epithelium, endothelium, and intestinal villus-like structures. (**iii**) 3D reconstructed confocal image and side view of a mock-infected gut-on-chip. (**iv**) 3D reconstructed confocal image and side view of the virus-infected intestinal model. SARS-CoV-2 infection was identified in the epithelial layer by the expression of the viral Spike protein. Reproduced from [127], with permission from Elsevier, 2021. (**B**) Breast cancer-on-chip for patient-specific efficacy and safety testing of CAR-T cells. CAR-T cells infiltrated the tumor aggregates and hampered their growth in a target-specific manner on the chip. Representative images of MDA-MB-231 aggregates or fibroblast spheroids on day 0 (before [CAR-]T cell treatment), and on days 1 and 8 after tumor-on-chip perfusion with control T cells or CAR-T cells. MDA-MB-231 tumor cells express GFP and are pseudocolored cyan. Fibroblasts—representing nonmalignant tissue as a control—are labeled with CellTracker 5-chloromethylfluorescein diacetate (CMFDA) and pseudocolored cyan. Control T cells and CAR-T cells were labeled with CellTracker deep red. The yellow dashed line marks each tumor aggregate or fibroblast spheroid. Microvascular endothelial cells (MvECs) were present in all chips. Scale bars, 200 μm. Reproduced from [130], with permission from Elsevier, 2024.

Pathogen-induced diseases exemplify the utility of OoCs. For instance, an intestinal infection-on-a-chip model was developed to study severe acute respiratory syndrome coronavirus 2 (SARS-CoV-2) [127]. The system incorporated intestinal epithelial cells (Caco-2, HT29) and endothelial cells (HUVECs) (Figure 4A). The intestinal channel was infected with a multiplicity of infection (MOI) of 1.0, and after 3 days, key pathological features were observed, such as damaged intestinal villi, disrupted tight junctions, and immune activation. The model demonstrated the potential of microfluidic devices in advancing our understanding of host–pathogen interactions and exploring therapeutic strategies.

Lifestyle-related diseases have also been successfully modeled. A liver-on-a-chip system was developed to study nonalcoholic steatohepatitis (NASH) [128]. The platform incorporated four primary liver cell types (hepatocytes, Kupffer cells, liver sinusoidal endothelial cells, and hepatic stellate cells), replicating key NASH features by exposing them to an overload of long-chain free fatty acids (FFAs) and LPS. This exposure led to lipid accumulation, hepatocellular ballooning, and inflammatory marker elevation. Additionally, the model demonstrated its preclinical value by evaluating Elafibranor (approved for treating Primary Biliary Cholangitis), which significantly reduced disease markers, highlighting the capacity of OoCs to bridge the gap between preclinical studies and human relevance in drug testing.

Neurodegenerative disorders, including Parkinson’s disease, have similarly benefited from OoC platforms. Dopaminergic cultures derived from iPSCs, humanized mice, and microfluidic neuronal systems were employed to evaluate CLR01, a molecular tweezer under pre-clinical investigation targeting alpha-synuclein (α-syn) aggregation [129]. The study reported consistent results across models, with CLR01 reducing the aggregation of α-syn and toxicity. In microfluidic devices, dopaminergic cultures were treated with recombinant α-syn oligomers, and CLR01 treatment prevented α-syn accumulation in neuronal cell bodies, underscoring the value of OoCs in translational neuroscience and therapeutic discovery.

In oncology, a breast cancer-on-a-chip model mimicking tumor-immune interactions and CAR-T cell therapies has been reported [130]. The system, developed with patient-derived tumor organoids and an endothelial barrier, enables immune cell infiltration, cytokine release, and monitoring for up to 8 days (Figure 4B). Its modular design and ability to evaluate CAR-T efficacy offer a personalized approach to optimizing therapies and addressing the limitations of traditional models.

Within the scope of precision medicine, bioprinted tumor models are valuable for recapitulating glioblastoma (GBM) pathophysiology. A GBM-on-a-chip was developed, integrating patient-derived tumor cells into a compartmentalized microenvironment that mimics tumor hypoxia [131]. The chip consists of a gas-permeable silicone chamber wall, an outer ring bioprinted with porcine brain-derived extracellular matrix (BdECM) bioink laden with endothelial cells (HUVECs), and an inner compartment containing GBM cell-laden BdECM bioink, forming a radial oxygen gradient. After 1–2 weeks of culture, the model successfully reproduced native tumor properties and patient-specific resistance to chemoradiation and temozolomide. Additionally, it facilitated drug combination screening, underscoring its potential as a precision oncology tool for therapy-resistant GBM patients.

In recent years, gut-on-a-chip systems have emerged as powerful platforms to model gastrointestinal diseases such as Inflammatory Bowel Disease (IBD) and, to a lesser extent, Irritable Bowel Syndrome (IBS), advancing our ability to replicate key pathophysiological features of the human intestine under controlled microfluidic conditions. For example, one study integrated human intestinal epithelial cells derived from organoids together with monocyte-derived macrophages within a microfluidic device; the epithelial cells under flow displayed enhanced polarization, and upon inflammatory stimulation, secreted CXCL10, IL-8, and CCL20 in patterns that aligned with dysregulated pathways observed in IBD patient tissue, underscoring the relevance of the model for drug screening and translational applications [132].

Similarly, a dual-channel microfluidic gut-on-a-chip designed to control oxygen gradients successfully simulated IBD-like epithelial barrier disruption induced by TNF-α and LPS; remarkably, the addition of Bifidobacterium bifidum mitigated barrier disruption and enhanced ZO-1 localization, suggesting potential mechanistic insights into microbe-host interactions in intestinal inflammation [133].

Beyond single-organ chips, multiorgan platforms have highlighted how intestinal inflammation might interface with other systems: one OoC lined with patient-derived colon epithelium and fibroblasts recreated hallmark IBD features—mucus loss, inflammation, and fibrotic changes—and revealed that peristalsis-mimicking mechanical deformation and sex-hormone exposure amplified disease phenotypes [134]. These examples illustrate how gut-on-a-chip models are transitioning from pure tissue mimics toward disease-relevant, patient-derived platforms capable of capturing the complexity of intestinal disorders.

The advancement of OoC platforms expands their potential in biomedical research. While many models focus on replicating a single organ, integrating multi-organ interactions enhances physiological relevance, especially for systemic diseases. Mature models must be scalable for high-throughput applications and provide consistent results.

### 4.3. Regenerative Medicine and Tissue Engineering

The convergence of 3D bioprinting and OoC platforms is revolutionizing tissue engineering and regenerative medicine by providing powerful tools for advancing tissue regeneration and stem cell differentiation. By combining the structural precision of bioprinting with the dynamic, physiologically relevant environments of OoC systems, researchers can mimic complex tissue architectures and microenvironments critical for guiding cellular behavior. These integrated technologies hold immense promise for creating implantable tissues and organs, offering transformative solutions for transplantation, and addressing the growing demand for viable alternatives to the organ shortage.

**Figure 5:**
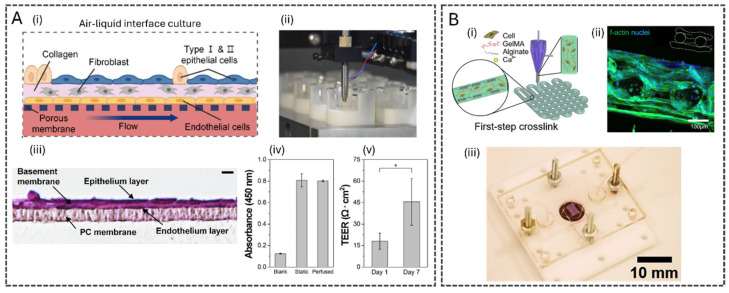
Three-dimensional Bioprinting and OoC in Regenerative Medicine and Tissue Engineering. (**A**) 3D Inkjet-Bioprinted Lung-on-a-Chip. (**i**) Schematic cross-section image of the alveolar barrier tissue on a chip. (**ii**) Image of the automatic tissue printing procedure from a single nozzle of the piezo-inkjet printer. (**iii**) H&E staining of the sectioned alveolar barrier tissue with a polycarbonate (PC) porous membrane. Scale bars: 10 μm. (**iv**) Comparison of the cell proliferation rates on the bioprinted tissues cultured on a well plate (static) and on a microfluidic device (perfused). (**v**) Measurement of the TEER value of alveolar barrier tissues cultured on a chip. Reproduced from [94], with permission from American Chemical Society, 2023. (**B**) 3D bioprinting allied with microfluidics for engineering endothelialized myocardium and heart-on-a-chip. (**i**) Schematic diagrams showing the bioprinting process with the alginate and GelMA bioinks. (**ii**) Cross-sectional view of a three-layer scaffold at Day 14, indicating the formation of the endothelium. (**iii**) Photograph of the bioreactor with an embedded bioprinted scaffold. Reproduced from [140], with permission from Elsevier, 2016.

Significant advances have been made in creating various tissues and organs, including skin [48,135], intestine [51], cartilage [136,137], and neural tissue, the latter considered one of the greatest challenges [138]. Notable examples include the bioprinted lung-on-a-chip platform, allowing the culture of 3D-bioprinted lung models with air-liquid perfusion [94]. The model maintains a three-layer structure with a functional alveolar barrier, fundamental for mimicking pulmonary tissue (Figure 5A). This versatile platform enables mass production and offers customization through bioprinting technology, providing a promising approach for personalized models.

A liver-on-a-chip platform was developed for long-term culture of 3D spheroids composed of hepatocellular carcinoma cells (HepG2/C3A) to assess drug toxicity. The bioreactor design allowed in situ monitoring of the culture environment, enabling direct access to the hepatic construct without compromising platform operation. This liver construct remained functional for 30 days, with acetaminophen treatment inducing a toxic response, demonstrating the platform’s potential for toxicity testing [139].

A hybrid strategy leveraging 3D bioprinting to fabricate endothelialized myocardium has been introduced [140]. They developed endothelialized myocardial tissues through bioprinting using alginate and GelMA-based bioinks, incorporating endothelial cells into the structures (Figure 5B). The endothelial cells were directly printed within microfibrous hydrogel scaffolds, where they migrated to form a confluent endothelial layer. This system was then integrated with cardiomyocytes, which spontaneously contracted in a synchronized manner. The resulting endothelialized myocardium was placed in a specialized microfluidic perfusion bioreactor, creating a platform for cardiovascular toxicity testing. Furthermore, the technique was successfully applied to human cardiomyocytes derived from induced pluripotent stem cells, offering the potential for regenerative medicine, drug screening, and disease modeling. These models illustrate how combining bioprinting, microfluidics, and stem cells can accelerate the creation of functional organs for various clinical applications.

These technological innovations offer vast potential to accelerate the development of therapeutic alternatives, with direct implications for treating various diseases and injuries. One of the areas that benefits most from these platforms is transplantation, with numerous experimental models already being conducted to test the viability of bioprinted tissues.

For instance, a 3D bioprinting platform has been reported to successfully produce human dermo-epidermal skin substitutes, which demonstrated not only structural resemblance to native skin but also promising functional properties, such as adequate barrier function and early vascularization in preclinical models [141]. These findings highlight the potential application of bioprinted skin grafts in treating severe burns, chronic wounds, and skin defects associated with conditions such as diabetes.

On the other hand, other bioprinting techniques have been employed to create autologous bone scaffolds for cranioplasty, significantly improving bone regeneration and osseointegration compared to traditional grafts. Additionally, cartilage repair strategies using mesenchymal stem cell-laden GelMA hydrogel in beagle dogs have shown enhanced chondrogenic differentiation, leading to improved mechanical properties and functional restoration of damaged cartilage [142].

Beyond musculoskeletal applications, advancements in neural tissue engineering have also demonstrated potential in treating neurological injuries. Studies involving neural stem cells in spinal cord injury models have reported enhanced neuronal survival, axonal regeneration, and partial functional recovery, suggesting a viable therapeutic approach for spinal cord injury rehabilitation [143].

Furthermore, islet organoids designed for diabetes treatment in mice have restored glucose homeostasis for extended periods, improving insulin secretion and reducing hyperglycemia without immunosuppressive therapy [144]. This approach could revolutionize diabetes management by offering a renewable source of functional pancreatic islets, addressing limitations associated with donor availability and immune rejection.

These studies illustrate the transformative potential of bioprinting and regenerative medicine, paving the way for personalized, effective, and durable therapeutic solutions for several complex medical conditions.

## 5. Challenges and Future Directions

Despite significant progress in 3D bioprinting and microfluidics, several technological challenges impose difficulties in their broader application in OoC platforms. One of the primary barriers is scalability [41,145]. Current bioprinting techniques may struggle to achieve reproducibility when transitioning from laboratory-scale to high-throughput industrial processes. This limitation extends to the fabrication of complex tissues with multiple cell types, where maintaining resolution and precision is critical for replicating native tissue architectures. For microfluidic systems, fabricating robust, leak-proof devices with intricate channel networks remains a technical hurdle, especially when scaling up production.

Another major limitation lies in ensuring long-term tissue viability. While advancements in bioink formulations and perfusion systems have improved cellular longevity, many bioprinted tissues suffer from inadequate nutrient and oxygen diffusion in larger constructs, resulting in necrosis at the core [146,147]. This issue highlights the ongoing need for enhanced vascularization strategies through bioprinting and complex microchannel integration.

Regulatory and standardization challenges further complicate the path to clinical and industrial adoption. The lack of standardized protocols for the design, fabrication, and testing of OoC platforms creates inconsistencies that hinder validation and reproducibility [148,149]. Furthermore, navigating the regulatory landscape for bioprinted and microfluidic systems is complex due to the novelty of these technologies and their intersection with multiple disciplines. Establishing clear quality control and safety assurance guidelines is essential to gain acceptance in pharmaceutical and clinical applications.

Emerging technologies offer promising solutions to overcome these barriers and drive the evolution of OoC platforms. One such advancement is 4D bioprinting, where time-dependent structural or functional changes are incorporated into printed constructs, allowing dynamic tissue maturation or responsiveness to environmental stimuli [150,151,152,153,154]. This approach can enhance tissue functionality and adaptability, particularly in mimicking complex physiological conditions.

AI and ML are poised to revolutionize tissue design and microfluidic modeling [155,156,157,158]. These data-driven tools can optimize bioink compositions, predict tissue behavior, and refine fluid dynamics within OoC systems, reducing the trial-and-error traditionally associated with experimental setups [157,158]. In particular, deep learning approaches, such as convolutional neural networks (CNNs) for image-based analysis of cell morphology and flow patterns, and recurrent neural networks (RNNs) or transformer architectures for modeling time-dependent physiological signals, offer powerful means to extract meaningful features from the high-dimensional, multimodal datasets generated by OoC platforms [159,160]. Such algorithms can automatically learn hierarchical representations of complex biological interactions, enabling improved prediction of tissue functionality, drug response, and disease progression [161]. Furthermore, integrating AI with robotic automation paves the way for fully automated bioprinting and microfluidic device fabrication, allowing standardized production, increased throughput, and real-time quality control of printed constructs [162].

A critical bottleneck for applying AI in OoC platforms is the limited availability of high-quality, standardized datasets [161]. Unlike conventional biomedical imaging or genomics datasets, OoCs generate multimodal, time-resolved, and highly heterogeneous data, spanning imaging, flow/pressure measurements, biochemical outputs, and functional read-outs, which pose major challenges for ML workflows. For example, one recent dataset for OoC image classification comprises only ~3000 images with binary quality labels (“good” vs. “bad”), and the authors note that data augmentation and expert consensus still left a model accuracy near 0.81 [163]. Moreover, although repositories such as the Organs-on-a-Chip Database (OOCDB) attempt to aggregate OoC datasets, they remain limited in scope and often lack harmonized metadata, consistent annotation standards, or accessible raw data structures [164]. Without sufficiently large, diverse, and well-annotated datasets, deep learning models risk overfitting, poor reproducibility, and limited generalizability. To advance AI-driven OoC systems, the community must prioritize data curation, adoption of common ontologies/metadata schemas, open-access sharing of raw and annotated data, and the development of cross-platform benchmark datasets, analogous to benchmark cohorts in medical imaging, to build trust and accelerate translational impact.

The prospect of integrated systems that combine bioprinting and microfluidics in a seamless workflow is also on the horizon [4,165]. Innovations in multimaterial bioprinters and hybrid manufacturing techniques are bringing us closer to OoC platforms that can be produced in a single-step [95,166]. These advances will significantly reduce production times and enhance the complexity of constructs, enabling more realistic tissue models for drug testing and disease modeling.

Hence, the future of 3D bioprinting and microfluidic OoC platforms lies in the convergence of multiple disciplines. Collaboration among bioengineering, materials science, and computational modeling experts is essential for addressing the multifaceted challenges associated with these technologies. For instance, materials scientists are developing novel bioinks with enhanced mechanical and biological properties [15,17], while computational modelers are creating sophisticated simulations to optimize flow dynamics and tissue behavior in microfluidic systems [108,109].

Integrating OoC platforms with complementary technologies such as biosensors, clustered regularly interspaced short palindromic repeats (CRISPR), and organoids represents a significant opportunity for innovation. Biosensors embedded within OoC systems can provide real-time data on tissue health, drug responses, and biomarker levels, enhancing the platforms’ utility in preclinical research [70,167,168,169]. CRISPR gene-editing technology enables the creation of genetically tailored tissues, offering personalized medicine applications and deeper insights into genetic diseases [170,171]. Similarly, incorporating organoids into OoC systems adds complexity and realism, bridging the gap between traditional 2D cell cultures and full-scale organ models [14,76,172]. Integrating patient-derived cells and organoids with 3D bioprinting and microfluidic-based OoC systems can transform personalized medicine by enabling highly tailored disease models and treatment strategies [173]. These technologies allow for the recreation of patient-specific tissue microenvironments, improving drug screening accuracy, predicting individual responses to therapies, and reducing reliance on animal models [174].

Interdisciplinary collaboration also extends to academia-industry partnerships. Pharmaceutical companies, biotechnology startups, and academic research labs must work together to translate laboratory innovations into scalable, regulatory-compliant solutions. Joint initiatives and consortia can accelerate progress by pooling expertise, resources, and funding. As these collaborations deepen, the resulting technologies will advance fundamental research and redefine the landscape of drug development, disease modeling, and regenerative medicine.

## 6. Conclusions

**Figure 6:**
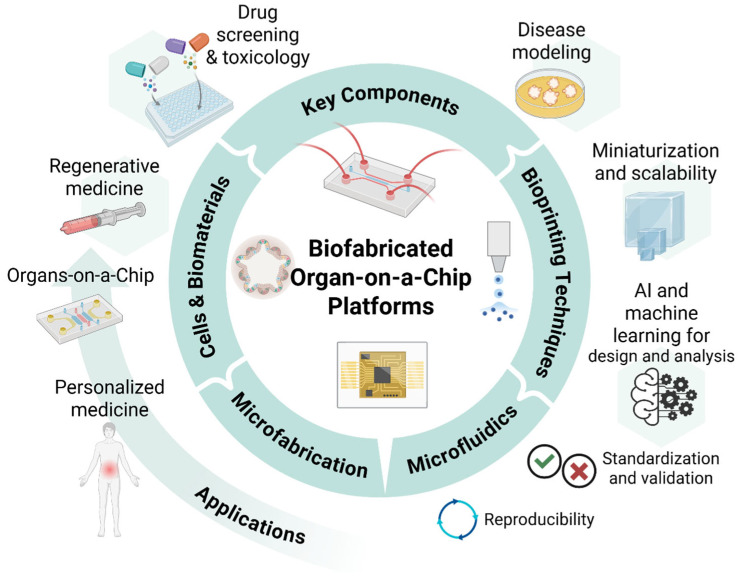
Overview of biofabricated OoC platforms and their applications. Advances in microfluidics, bioprinting, and biomaterials have enabled the development of OoC systems replicating human tissue function. These platforms support applications in drug screening, disease modeling, and personalized medicine. Emerging technologies such as AI, 4D bioprinting, and biosensors are driving improvements in scalability, reproducibility, and predictive power. Created in BioRender. Figueira, A. (2025) https://BioRender.com/2rmd26d.

The rapid advancements in 3D bioprinting and microfluidics have redefined the possibilities in tissue engineering and OoC platforms. Key technological breakthroughs in high-resolution bioprinting, advanced bioink development, and dynamic microfluidic systems have enabled the creation of physiologically relevant tissue models. These platforms mimic human organs’ complex architecture and functionality, providing unparalleled opportunities for drug discovery, disease modeling, and personalized medicine (Figure 6). Integrating vascularized tissues and perfusable constructs has been particularly transformative, addressing critical challenges such as nutrient and oxygen diffusion in engineered tissues.

Looking ahead, several key areas hold the promise of transformative impact. The rise of 4D bioprinting is expected to enhance the functionality of OoC systems by introducing constructs that adapt and evolve. Advances in AI-driven design and automation will streamline the development of complex bioprinted tissues and microfluidic devices, enabling higher throughput and greater reproducibility. Furthermore, integrating complementary technologies, such as biosensors, CRISPR, and patient-derived organoids, is pushing the boundaries of OoC capabilities, creating more predictive and tailored platforms for precision medicine.

Despite current limitations, the progress in this field signals a future where bioprinting and microfluidics converge seamlessly to deliver scalable, cost-effective, and clinically relevant solutions. These innovations will undoubtedly redefine drug development, toxicology testing, regenerative medicine, and transplantation, paving the way for a new era of biomedical engineering. By addressing existing challenges and fostering interdisciplinary collaboration, researchers will unlock the full potential of these technologies, transforming the landscape of healthcare and research.

## Figures and Tables

**Table 1:** Representative quantitative parameters reported in recent 3D bioprinting and microfluidic OoC studies.

Platform Type	Technique/Model	Key Quantitative Parameters	Reference
3D bioprinting	Volumetric (VBP) hepatic organoids	Resolution ≈ 50 µm; viability > 90%; albumin synthesis ↑ 2.3× vs. 2D	[32]
3D bioprinting	Extrusion-based cardiac patch	Perfusable channels 250 µm Ø; synchronous contraction > 90% cells	[44]
3D bioprinting	Intestine-on-a-chip	Flow 15 nL min^−1^; MUC-2 expression ↑ 3× vs. static; villi ≈ 300 µm	[47]
Microfluidic OoC	BBB-on-a-chip	Flow 16 µL min^−1^; shear ≈ 4 dyn cm^−2^; TEER ≈ 250 Ω·cm^2^	[78]
Microfluidic OoC	Gut–liver chip (NAFLD model)	Flow 15 nL min^−1^; lipid accumulation ↑ after 7 days; albumin ↑ 1.5×	[77]
Multi-OoC	Liver–heart system	Albumin ≈ 15 µg mL^−1^ (day 7); spontaneous beating > 70 bpm	[87]

## Data Availability

No new data were created or analyzed in this study. Data sharing is not applicable to this article.
